# A Spatiotemporal Brain Network Analysis of Alzheimer’s Disease Based on Persistent Homology

**DOI:** 10.3389/fnagi.2022.788571

**Published:** 2022-02-09

**Authors:** Jiacheng Xing, Jiaying Jia, Xin Wu, Liqun Kuang

**Affiliations:** ^1^School of Data Science and Technology, North University of China, Taiyuan, China; ^2^Department of Computer Science, University of Birmingham, Birmingham, United Kingdom

**Keywords:** Alzheimer’s disease, brain network, functional magnetic resonance imaging, dynamic functional connectivity, persistent homology, sliding window

## Abstract

Current brain network studies based on persistent homology mainly focus on the spatial evolution over multiple spatial scales, and there is little research on the evolution of a spatiotemporal brain network of Alzheimer’s disease (AD). This paper proposed a persistent homology-based method by combining multiple temporal windows and spatial scales to study the spatiotemporal evolution of brain functional networks. Specifically, a time-sliding window method was performed to establish a spatiotemporal network, and the persistent homology-based features of such a network were obtained. We evaluated our proposed method using the resting-state functional MRI (rs-fMRI) data set from Alzheimer’s Disease Neuroimaging Initiative (ADNI) with 31 patients with AD and 37 normal controls (NCs). In the statistical analysis experiment, most network properties showed a better statistical power in spatiotemporal networks than in spatial networks. Moreover, compared to the standard graph theory properties in spatiotemporal networks, the persistent homology-based features detected more significant differences between the groups. In the clustering experiment, the brain networks on the sliding windows of all subjects were clustered into two highly structured connection states. Compared to the NC group, the AD group showed a longer residence time and a higher window ratio in a weak connection state, which may be because patients with AD have not established a firm connection. In summary, we constructed a spatiotemporal brain network containing more detailed information, and the dynamic spatiotemporal brain network analysis method based on persistent homology provides stronger adaptability and robustness in revealing the abnormalities of the functional organization of patients with AD.

## Introduction

Alzheimer’s disease (AD) (Scheltens et al., 2021) is one of the classic chronic neurodegenerative diseases, which is considered as a common form of dementia. With the progress of this disease, it gradually spread to different brain regions, thus greatly affecting the patient’s daily life (Patterson, 2018). Currently, the resting-state functional MRI (rs-fMRI) (Engels et al., 2017) has become an important technology to explore brain functional networks. A lot of early brain functional alterations related to AD pathophysiology have been reported using various brain network analysis methods (Hallquist and Hillary, 2018; Marquez and Yassa, 2019; Mill et al., 2020). Most of these methods assume that the functional connectivity is constant during the brain imaging process, which may ignore some key time-varying information of the brain network caused by healthy brain or nervous system diseases. Therefore, it is necessary to study dynamic functional connectivity based on temporal dynamics (Preti et al., 2017).

Currently, the main methods used in the dynamic functional connectivity analysis include sliding window analysis, clustering analysis, time-frequency coherence analysis, and dynamic graph theory analysis (Preti et al., 2017). Among them, a large number of studies (Chen et al., 2016, 2021; de Vos et al., 2018; Lei et al., 2021) have adopted the time-sliding window approach to analyze the time-varying domain of functional connectivity. In this way, an entire rs-fMRI time series is divided into multiple subseries. For each segment, a functional connectivity network is constructed to measure the short-term correlation between the brain regions. Thus, all networks constructed from all segments describe the dynamics of the short-term functional connectivity over time. Further, the volatility between specific time points or time windows is observed by some network metrics. Specifically, a dynamic graph theory-based analysis method has developed a series of network metrics (Hallquist and Hillary, 2018; Sporns, 2018) to detect the differences of brain network structures over time in rs-fMRI research. Using the method of sliding window, more differences between the groups in a dynamic brain network can be observed, which cannot be seen in a static brain network. Multiple time windows provide more temporal evolution information for the brain network analysis. However, the spatial evolution information is missing as the brain network is measured on a single fixed spatial scale.

Recently, persistent homology (Edelsbrunner and Harer, 2010) is an important research tool of topological data analysis, which focuses on exploring topological invariants with the increase of spatial scales. In the process of spatial scale change, the topological feature with a shorter duration is considered as noise, and the feature with a longer duration is used to represent an essential feature (Giusti et al., 2016). Compared with the traditional graph theory method, it can better reveal topological changes in space and avoid the problem of scale threshold selection. Some studies have successfully applied the persistent homology technology to analyze the brain network structure of AD (Kuang et al., 2019a, 2020b), epilepsy (Choi et al., 2014), autism spectrum disorder, attention-deficit hyperactivity disorder (Lee et al., 2012, 2017), etc. In our prior study on AD brain networks (Kuang et al., 2019a), we have proposed an integrated persistent feature (IPF) based on persistent homology that achieves holistic descriptions of spatial dynamics of the brain network. We have also found that the IPF is more robustness than graph theory-based metrics in our prior studies (Kuang et al., 2019a,b, 2020a,b). However, all existing studies on persistent homology only focus on the feature invariants in the process of spatial dynamics, but no literature studies have reported the influence of the change of time window on feature invariants.

In summary, there are a few available frameworks that can simultaneously quantify the brain network structure both in the time domain and in the spatial domain. As the time-sliding window approach can produce multiple dynamic time windows for studying the characteristics of time-varying connectivity, and the persistent homology method has been verified to be good at measuring the characteristics of space-varying connectivity, we propose a novel method combining spatial scale filtrations of persistent homology with temporal sliding windows. We hypothesize that such generated spatiotemporal network dynamics may improve the performance of detecting AD-induced topological changes in the rs-fMRI analysis, and the derived network properties based on persistent homology may be used as the potential biomarkers for AD imaging.

In this paper, we develop a persistent homology-based method by combining multiple temporal windows and spatial scales to study the spatiotemporal evolution of brain functional networks. Specifically, a time-sliding window method is performed to establish a spatiotemporal network and the persistent homology-based features of such a network are obtained. In our experiments, we evaluate our proposed method using rs-fMRI data set with 31 patients with AD and 37 normal controls (NCs). We test whether the proposed method provides stronger adaptability and robustness in revealing the abnormalities of the functional organization of patients with AD.

## Materials and Methods

**FIGURE 1:**
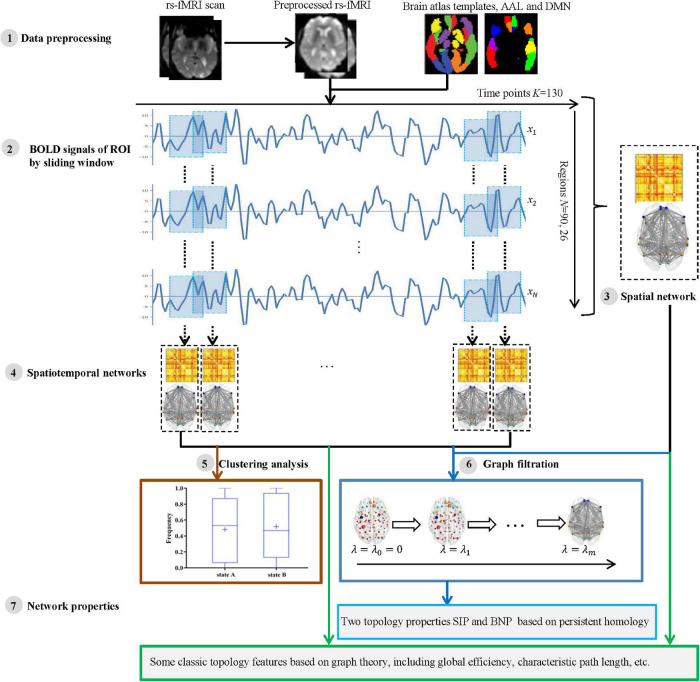
The pipeline of measuring brain network topological structure based on graph theory and persistent homology using resting state functional MRI (rs-fMRI) data from Alzheimer’s Disease Neuroimaging Initiative (ADNI).

For each participant, the processing and analysis pipeline is summarized in Figure 1. Initially, the rs-fMRI data were acquired and preprocessed using well-known toolboxes (Step 1). Then, each subject’s blood oxygen-level dependent (BOLD) signals within each region of interest (ROI) were obtained and divided into some segments by moving a sliding window over it (Step 2). For each subject, we then constructed a spatial network (Step 3) from the entire BOLD signals, as well as a series of spatiotemporal networks (Step 4) from the divided signal segments. Further, we applied a clustering analysis to spatiotemporal networks to verify whether the network structures of AD and NC are different (Step 5). Finally, we measured the network topology properties (Step 7) of each subject using the two kinds of methods, i.e., traditional graph theory and novel persistent homology based on graph filtration (Giusti et al., 2016) (Step 6). The specific steps are described in the following subsections.

### Data Acquisition and Preprocessing

The data used in the preparation of this article were obtained from the Alzheimer’s Disease Neuroimaging Initiative (ADNI) database^1^ (Jack et al., 2008; Jagust et al., 2010), which are used to measure the progression of mild cognitive impairment (MCI) and early AD. The rs-fMRI data set was acquired from ADNI-2 in this study. Specifically, the 3.0T Philips MRI scanner was used to scan the brain of the subjects, and a high-resolution rs-fMRI three-dimensional image was acquired by an echo plane imaging (EPI) sequence. The acquisition parameters are as follows: echo time (TE) = 30 ms, turning angle = 80°, slice thickness = 3.3 mm, slice number = 48, matrix size = 64 × 64, repetition time (TR) = 3,000 ms, and time point volume = 140.

In this study, we used the commonly available rs-fMRI data of the ADNI database and screened the subjects’ age, education level, and gender. Thus, there were a total of 68 AD and NC studies. Then, spm12^2^ and DPARSF toolboxes^3^ (Yan and Zang, 2010) were applied for rs-fMRI data preprocessing. The specific steps are as follows.

Firstly, for each subject, the first 10 time points of rs-fMRI images were discarded to improve the signal-to-noise ratio and achieve signal stability. The remaining 130 time points were used for further analysis.

Secondly, the time correction was applied according to the acquisition time difference between the scanning layers. The slice at TR/2 time point was used as the reference scanning layer, and other scanning layers were aligned to it to ensure that the acquisition of each slice corresponds to the same time point.

Further, a spatial correction was applied. Each subject’s functional images were aligned to the Montreal Institute of Neurology (MNI) space according to its structural image (T1 image).

Then, spatial smoothing with a Gaussian kernel of 4 × 4 × 4 mm full width at half maximum, linear trend removal, band-pass filtering (0.01–0.08 Hz), and global signal regression were carried out in turn.

Finally, a covariate regression analysis was used to eliminate the signal fluctuation caused by a cerebrospinal fluid signal, white matter signal, and head movement.

As there is still a controversy on whether to eliminate the whole brain signal, this study did not regress the whole brain signal. At the end of the experiment, the data of head displacement more than 1 mm or of head-motion rotation more than 1° were excluded.

### Construction of Brain Networks

We constructed the two modes of brain network, i.e., spatial network and spatiotemporal network. A spatial network was established according to the brain partition template on spatially anatomical labeling, while a spatiotemporal network was constructed by sliding a time series window.

#### Construction of Spatial Network

According to standard automated anatomical labeling (AAL) atlas (Tzourio-Mazoyer et al., 2002), the whole brain is spatially divided into 90 ROIs. In this paper, we also studied another subnetwork of AAL with 26 ROIs, i.e., default mode network (DMN). After image preprocessing, the average rs-fMRI time series (a total of 130 time series, *K* = 130) of each brain region were summarized, and the Pearson correlation coefficient between each pair of ROIs was defined as its functional connectivity. Therefore, the spatial brain network of each subject was constructed and represented as an adjacency matrix with a size of 90 × 90 or 26 × 26 (*N* = 90 or 26). Specifically, each ROI represents a node, and the edge weight between ROI *i* and *j* is defined based on Pearson correlation, i.e.,


(1) $$w_{ij}=1-\frac{cov\left(x_{i},x_{j}\right)}{\sigma_{x_{i}}\sigma_{x_{j}}}=1-\frac{\sum_{k=1}^{N}\left(x_{ik}-{\bar{x}}_{i}\right)\left(x_{jk}-{\bar{x}}_{j}\right)}{\sqrt{\sum_{k=1}^{N}{\left(x_{ik}-{\bar{x}}_{i}\right)}^{2}}\sqrt{\sum_{k=1}^{N}{\left(x_{jk}-{\bar{x}}_{j}\right)}^{2}}}\;,$$

where *x*_*i*_ = (*x*_*i*1_, *x*_*i*2_, ⋯, *x*_*iN*_) and *x*_*j*_ = (*x*_*j*1_, *x*_*j*2_, ⋯, *x*_*jN*_) are the time series of BOLD signals at the *i*th and *j*th ROI, respectively.

#### Construction of Spatiotemporal Network Based on Sliding Time Window

**FIGURE 2:**
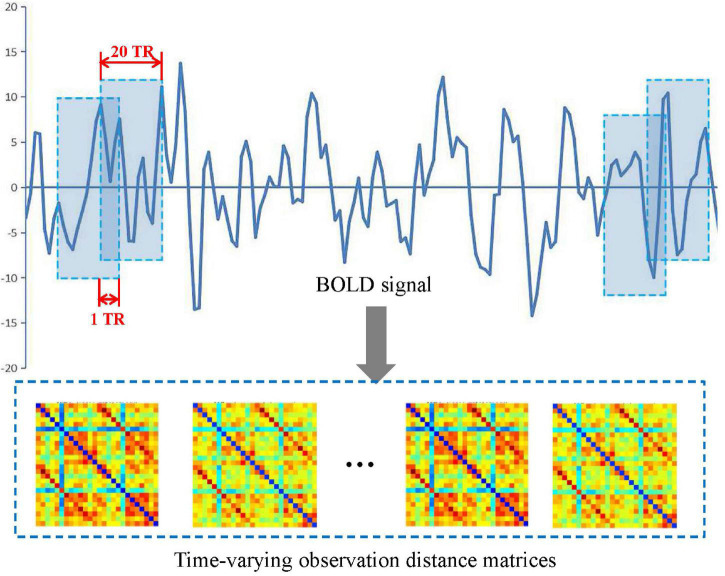
Construction of a spatiotemporal brain network by the sliding window method.

In this study, a sliding window method (Chen et al., 2016, 2021; de Vos et al., 2018; Lei et al., 2021) was performed to establish a spatiotemporal network as shown in Figure 2. A detailed construction process is described as follows.

First, a conical window was created by convoluting a sliding time series window with a Gaussian kernel (σ = 3TR). Compared with the traditional rectangle, the conical window has the advantage of reducing the weight of boundary time points when the subsequence window is sliding. The sliding window width *W* was set to 20TR (60 s) because previous studies have shown that a window width of 30–60 s is able to successfully capture resting-state functional connectivity fluctuations (Preti et al., 2017).

Then, for each subject, we moved the sliding window gradually in the step of one TR each time and thus produced (*K − W* + *1*) subsequences of time windows. Here, *K* = 130 is the length of the time series.

Further, for each time window *i*, an observation distance matrix *C*_*i*_ (*i* = 1, 2, *K* − *W* + 1) was generated based on Pearson correlation according to Equation (1), and the size of matrix is *N* × *N* (*N* is the number of ROI, *N* = 90 or 26).

Thus, each subject had a series of time-varying matrices, which could capture the dynamic changes of functional connectivity during the resting-state scanning. A time-varying matrix is used as a spatiotemporal network (see step 4 in Figure 1).

Finally, to quantify fluctuations in the connectivity time courses, some summary measures have been used, such as their SD (Preti et al., 2017; de Vos et al., 2018) or covariance (Chen et al., 2016). In this study, for each subject, we calculated a SD matrix across all time-varying matrices to quantitatively estimate the stability of functional connectivity over time.

### Spatiotemporal Network Clustering Analysis

In this study, we propose a novel method based on persistent homology to detect the differences of dynamic functional networks between AD and NC groups. However, the mostly applied strategy in this area is the state extraction of dynamic functional connectivity through *k*-means clustering (Preti et al., 2017). To verify the effectiveness of our proposed method, we also use the traditional method to conduct experiments on spatiotemporal networks. Specifically, we used *k*-means clustering (de Vos et al., 2018) to capture the state changes of functional connectivity in a dynamic time series, and then analyzed the differences between the groups of all patients with AD and NC subjects in the identical states of dynamic functional connectivity. The *k*-means clustering method uses a distance function to cluster data into different clusters in an iterative manner. It clustered the time-varying matrices of all sliding windows of all individuals into *k* clustering states. The similarity within the cluster is very high, while the similarity between the clusters is extremely low. In this study, the *k*-means algorithm was applied to a series of time-varying matrices according to its Manhattan distance (Allen et al., 2014).

We further observed each clustering state from two aspects, window ratio and average residence time using the DynamicBC toolbox (Liao et al., 2014). Specifically, the window ratio is calculated as the proportion of staying in a given state window, and the average residence time represents the number of consecutive windows belonging to a state.

### Network Properties

In this study, network measurement methods based on graph theory and persistent homology were used to measure brain functional organization.

#### Graph Theory-Based Network

Graph theory analysis methods (Sporns, 2018) have been widely used in the study of brain network topology structure. This paper mainly used the currently widely used graph theory indicators. We measured the global and local transmission capabilities of the network (global efficiency and local efficiency) (De Pasquale et al., 2016), the shortest path between the brain regions (characteristic path length) (Brier et al., 2014), the importance of nodes (eigenvector centrality) (Binnewijzend et al., 2014), the degree of clustering of nodes (clustering coefficient) (Rauchmann et al., 2021), the characteristics of the small-world network (Watts and Strogatz, 1998), and the minimum eccentricity of nodes (network radius) (Fujita et al., 2017). As these graph theory indicators usually measure the brain network on a fixed scale, the brain network needs to be filtered before measuring its structure. The statistical analysis of connectivity has been commonly used to filter the network. Specifically, we deleted the edges that were not significantly connected in the brain network (*p* > 0.05) to obtain a sparse structure (Smith and Nichols, 2009; Wang et al., 2013). Then, we applied a brain connectivity toolbox^4^ (Rubinov and Sporns, 2010) on the filtered brain network to calculate these graph theory-based indicators.

#### Persistent Homology-Based Network Properties

Persistent homology (Edelsbrunner and Harer, 2010) is a mathematical concept from the algebraic topology. The typical approach of persistent homology is Betty number plot (BNP) (Lee et al., 2012; Giusti et al., 2016), which can distinguish persistent features from noise in graph filtering and is considered as a useful feature descriptor. It has been successfully used in brain network research based on fluorodeoxyglucose-positron emission tomography (FDG-PET) and MRI data in some neurodegenerative diseases (Lee et al., 2012, 2017; Choi et al., 2014). In our previous research, we proposed an IPF (Kuang et al., 2019a), which integrates a connected component aggregation cost with BNP to realize the spatial evolution of the overall graph. The IPF is defined as follows:


(2) IPFλi={m-im(m-1)∑k=i+1m-1λk,  0≤i≤m-2        0.        i=m-1  ,

where *m* is the maximum number of connected components, λ is a series of filtration values (λ*_0_* = *0* < λ*_1_* < λ*_2_* < … < λ*_*m*–1_*) generated by all edge weights of the maximum connected components of the brain network (see step 6 in Figure 1).

Further, the medical field tends to use a single indicator to measure biomarkers. Because IPF is a monotonically decreasing convergence function, the slope of IPF (SIP) is used as an index to quantify the dynamic research of AD brain network. We have successfully applied the SIP to AD brain network analysis in our prior studies (Kuang et al., 2019a,b, 2020a,b). Our opensource code of persistent homology can be downloaded at http://gsl.lab.asu.edu/software/IPF.

### Statistical Test

A two-sample *t*-test was performed on the brain network properties based on graph theory and persistent homology, as well as clustering states. *p* < 0.05 indicates that the difference between the two groups is statistically significant. We used Matlab (R2017a) for statistical test.

## Results

### Demographic Information

The experimental participants were obtained from ADNI-2.^5^ Details of demographic characteristics are shown in Table 1. There were 31 AD subjects aged 60–90 years old in rs-fMRI data set. To conduct a comparative study with the patient group, 37 matched NC subjects were selected in this study. Diagnostic classification was made by ADNI investigators using the established criteria. The Clinical Dementia Rating (CDR) global score of any AD patient was no less than 1, while that of NC subject was 0. There are no significant differences in age, education, and gender between the groups. However, there is a significant difference in Mini-Mental Status Examination (MMSE) scores (Folstein et al., 1975). In addition, the displacement of head movement in any subject’s image is less than 1 mm, and the rotation angle in any direction is less than 1°.

**TABLE 1:** Demographic information of experimental subjects.

	AD (*n* = 31)	NC (*N* = 37)	*p*-value
Age	74.0 ± 6.1	74.1 ± 6.2	0.7486
Education	15.4 ± 3.9	16.1 ± 3.6	0.4267
Gender (male/female)	16/15	15/22	0.6033
MMSE	22.8 ± 3.4	28.8 ± 1.6	0.0015
CDR score	≥ 1	0	–

*Data are presented as mean ± SD.*

*AD, Alzheimer’s disease; NC, normal control; MMSE, Mini-Mental State Examination; CDR, clinical dementia rating.*

### Whole Brain Network Properties

In this study, the properties based on graph theory and persistent homology methods were used for the whole brain network analysis using AAL atlas. Their statistical differences between groups are shown in Table 2 using a two-sample *t*-test.

**TABLE 2:** Statistical *p*-values of different network properties between Alzheimer’s disease (AD) and normal control (NC) groups in automated anatomical labeling (AAL).

Network mode	Graph theory-based properties						Persistent homology-based properties		
	GE	LE	CPL	EC	CC	SW	NR	SIP	BNP
Spatial network	0.075	0.828	0.484	0.092	0.357	0.763	0.049*	0.008**	0.041*
Spatiotemporal network	0.230	0.642	0.518	0.086	0.327	0.119	0.311	0.002**	0.003**

*p < 0.05; **p < 0.01.

*GE, global efficiency; LE, local efficiency; CPL, characteristic path length; EC, eigenvector centrality; CC, clustering coefficient; SW, small-world attribute; NR, network radius; SIP, slope of integrated persistent feature plot; BNP, Betty number plot.*

In the network measurement using graph theory-based properties, the network radius showed significant differences between patients with AD and subjects with NC (*AD*: 6.17 ± 2.63; *NC*: 5.97 ± 2.64, *p* = 0.0493) in a spatial network. However, other network properties based on the graph theory could not detect any significant differences between the groups either in a spatial or spatiotemporal network.

Then, in the network measurement using persistent homology-based properties, the SIP index showed very significant differences between the groups in a spatial network (*AD*: − 0.67 ± 0.13; *NC*: − 0.75 ± 0.10, *p* = 0.008 < 0.01), so does the BNP index (*AD*: − 257.16 ± 43.18; *NC*: − 283.12 ± 56.82, *p* = 0.041 < 0.05). Moreover, in the spatiotemporal network measurement, the group difference of BNP is more significant (*AD*: − 718.53 ± 84.92; *NC*: − 793.13 ± 109.96, *p* = 0.0030, much less than 0.041) than in a spatial network, and so is SIP (*AD*: − 0.52 ± 0.11; *NC*: − 0.59 ± 0.09, *p* = 0.002 < 0.008).

To sum up, the differences of persistent homology indicators between AD and NC are significantly greater than those of graph theory indicators. Moreover, their group differences in a spatiotemporal network are more significant than those in a spatial network. It suggests that a brain spatiotemporal network analysis based on persistent homology is more likely to explore potential biomarkers.

### Brain Default Mode Network Properties

Default mode network is a functional subnetwork with the strongest variability in the study of AD, which contains the most obvious areas of brain atrophy. In this study, 26 areas (Vriend et al., 2018) in AAL were identified as the ROIs of DMN. The statistical differences of network properties between the groups in the DMN atlas are shown in Table 3.

**TABLE 3:** Statistical *p*-values of different network properties between AD and NC groups in a default mode network (DMN).

Network mode	Graph theory-based properties						Persistent homology-based properties		
	GE	LE	CPL	EC	CC	SW	NR	SIP	BNP
Spatial network	0.058	0.960	0.619	0.009**	0.230	0.074	0.164	0.004**	0.049*
Spatiotemporal network	0.177	0.447	0.353	0.017*	0.145	0.007**	0.547	0.003**	0.001**

*p < 0.05; **p < 0.01.

*GE, global efficiency; LE, local efficiency; CPL, characteristic path length; EC, eigenvector centrality; CC, clustering coefficient; SW, small-world attribute; NR, network radius; SIP, slope of integrated persistent feature plot; BNP, Betty number plot.*

In the study of DMN based on the graph theory method, there is no significant difference in the small-world attribute in the spatial DMN (*p* > 0.05), but it shows a very significant difference in the spatiotemporal network (*AD*: 1.03 ± 0.02; *NC*: 1.02 ± 0.01, *p* = 0.007), suggesting a small-world attribute could detect subtle time variability in the spatiotemporal DMN. In addition, it can be seen that the eigenvector centrality has very significant differences between the groups in both the spatial network (*AD*: 0.1046 ± 0.01; *NC*: 0.1047 ± 0.01, *p* = 0.009) and spatiotemporal network (*AD*: 0.1946 ± 0.01; *NC*: 0.1950 ± 0.01, *p* = 0.017), while there is no difference in the whole brain study.

Then, in the study of DMN based on persistent homology, both SIP and BNP detected significant differences between the groups in spatial and spatiotemporal networks. Especially in the spatiotemporal DMN, the SIP (*AD*: − 0.69 ± 0.12; *NC*: − 1.46 ± 0.10, *p* = 0.003) and BNP (*AD*: − 171.41 ± 32.24; *NC*: − 198.84 ± 34.08, *p* = 0.001) have very significant group differences.

In general, the spatiotemporal DMN properties based on persistent homology performed the best. Research on the persistent homology of spatiotemporal DMN is more likely to distinguish AD from NC, and it is more likely to explore the potential biomarkers for AD imaging.

### Spatiotemporal Network Clustering Results

**FIGURE 3:**
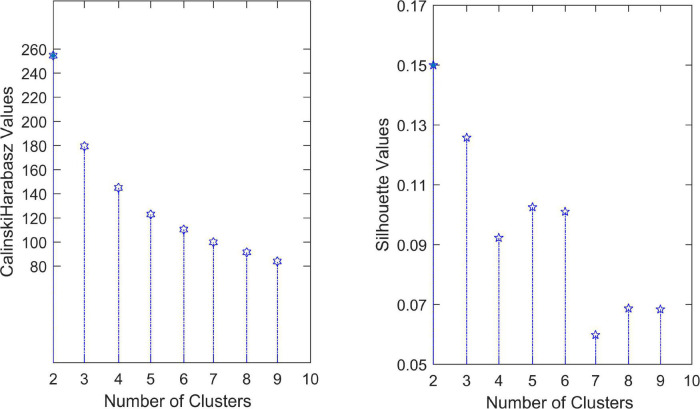
Optimization for the number of clusters.

**FIGURE 4:**
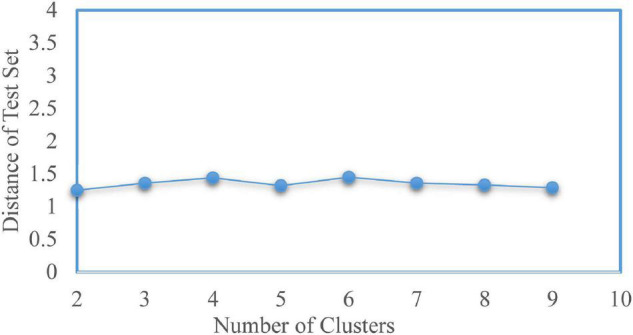
Cross-validation to determine the number of clusters.

To determine the optimal number of clusters *k*, we took *k* from 2 to 9 and repeated the test 100 times for each value. The experimental result is shown in Figure 3, where the solid point represents the best number of clusters. Here, the most suitable number of clusters was *k* = 2. The Silhouette score and Calinski–Harabasz index were used to evaluate the effectiveness of the clustering results. We further check the optimal number of clusters using a fivefold cross-validation. The average distance of test sets was calculated while selecting different cluster numbers, as shown in Figure 4. The error distance reached the minimum when the cluster number was 2, suggesting that the optimal value of *k* was 2 again. Thus, the AD and NC subjects’ varying-time matrices of sliding windows were clustered into two highly structured functional connectivity states separately.

**FIGURE 5:**
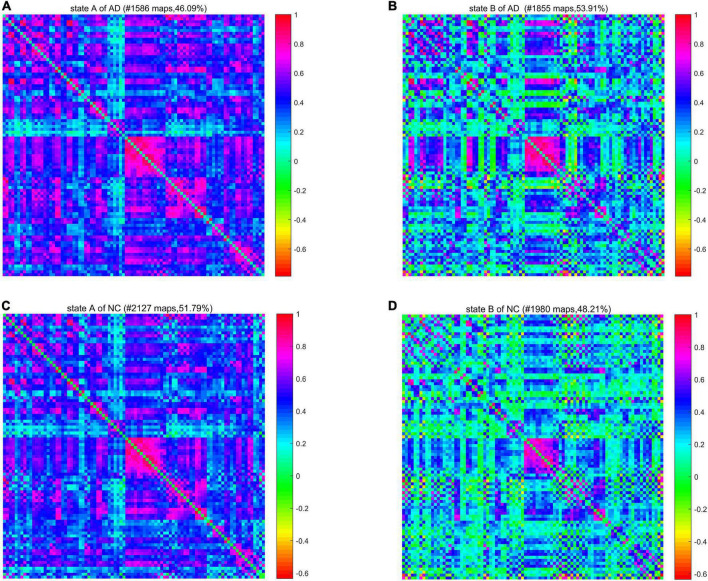
The cluster center diagram of spatiotemporal automated anatomical labeling (AAL) networks for Alzheimer’s disease (AD) and normal control (NC).

**FIGURE 6:**
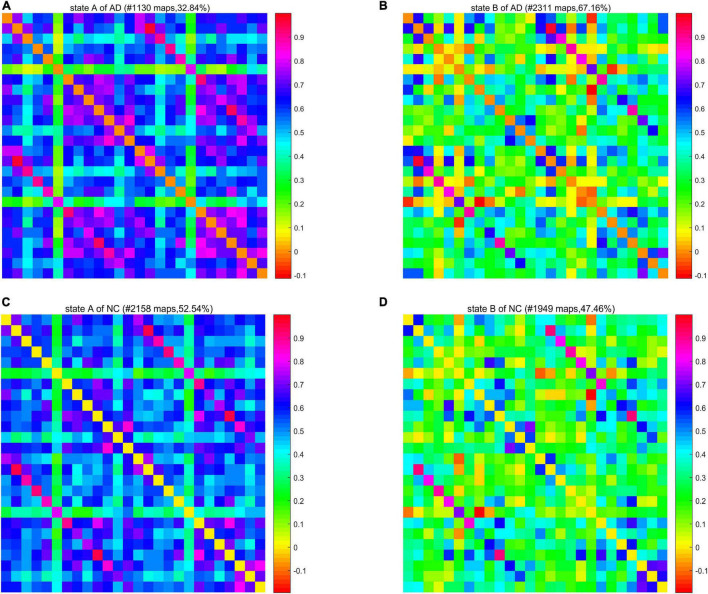
The cluster center diagram of spatiotemporal default mode networks (DMNs) for AD and NC.

We calculated a specific functional connectivity matrix (i.e., cluster center) for all windows in each state, and each element in the matrix was the median value of the corresponding element belonging to a state in all window matrices. The cluster center diagrams of the whole brain network and DMN are shown in Figures 5, 6, respectively, where maps correspond to the number of windows in a state, and the color bar represents the Pearson correlation coefficient value. It can be seen that state A is a stronger connection state, and state B is a sparser one. In the whole brain network clustering (see Figure 5), the window ratio of patients with AD in state A is reduced by about 5% compared with NC subjects, while it increases accordingly in state B. Similarly, in the DMN clustering (see Figure 6), compared with NC subjects, the window ratio of patients with AD in state B (*AD*: 0.67 ± 0.05; *NC*: 0.47 ± 0.06, *p* < 0.05) is significantly higher.

**FIGURE 7:**
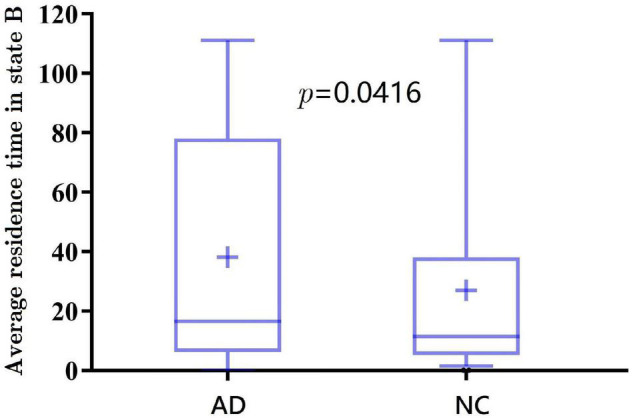
The average residence time of spatiotemporal AAL networks for AD and NC.

**FIGURE 8:**
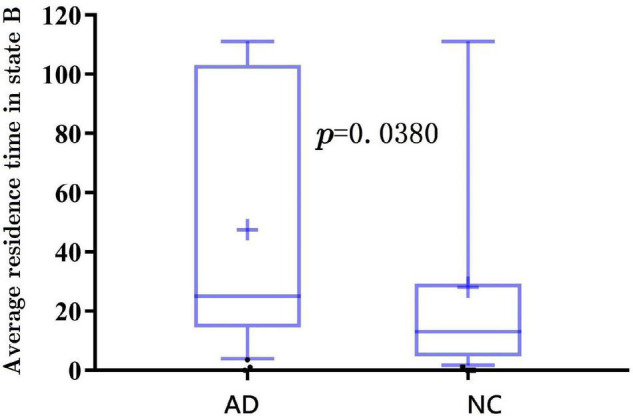
The average residence time of spatiotemporal DMNs for AD and NC.

Further, the results of average residence time are shown in Figures 7, 8. It is calculated by averaging the number of consecutive windows before changing from one to another state. The number of consecutive windows of AD in state B is relatively large, either in the whole brain network (see Figure 7, AD: 40 ± 8 windows; NC: 27 ± 5 windows, *p* < 0.05), or in DMN (see Figure 8, *AD*: 47 ± 8; *NC*: 28 ± 6, *p* < 0.05).

In summarily, the clustering results of the DMN subnetwork were similar to those of the whole brain network, and the difference between the AD and NC group was more significant. The brain network of patients with AD is more likely to stay in state B, a weak connection state.

## Discussion

### Present Findings

This paper studies the differences of spatiotemporal network dynamics between patients with AD and NCs in the whole brain network and its subnetwork DMN. There are three main findings in this study.

First, the spatiotemporal dynamics method combining a sliding window and persistent homology performs better than existing methods in the AD brain network analysis, and the persistent homology-based measures can be used as a potential biomarker. In our experiment, most of the network properties were more powerful in the spatiotemporal brain network than in the spatial brain network, particularly two persistent homology-based properties. Both SIP and BNP detected significant group differences (*p* < 0.01) in a spatiotemporal network after introducing time-varying information, in both the whole brain network and DMN subnetwork. In addition, in the DMN, the small-world attribute of a spatiotemporal network (*p* < 0.01) is significantly different from that of a spatial network (*p* > 0.05). In general, the brain spatiotemporal network could provide a more subtle temporal variability than a spatial network.

Second, in the study on the AD brain network, the DMN is a more suitable network than the whole brain network. The difference between groups of most network properties in DMN was more apparent than that in the whole brain network. In the DMN measurement using the graph theory method, the eigenvector centrality detected very significant differences between the groups in both spatial and spatiotemporal networks, while there was no difference in the whole brain study. Similarly, there was no significant difference in the small-world attribute in the whole brain network, but there was a very significant difference in the spatiotemporal DMN. Overall, the DMN is more likely to distinguish AD from NC, and it is more likely to explore the potential biomarkers for AD imaging.

Finally, the clustering results show that AD tends to be in the weak connection state. The window ratio and the average residence time in the weak connection state were relatively higher for patients with AD than those of NC. It may be due to the fact that patients with AD have not established a firm connection.

### Experimental Result Analysis of Proposed Method

As the persistent homology-based features are monotonically decreasing convergence functions according to filtration values, both the network properties BNP and SIP can be considered as the information diffusion rate or convergence rate in the process of reaching a fully connected component. In this study, in both the whole brain network and DMN, the values of the BNP group and the SIP group showed the same pattern of | AD| < | NC|, with a significance in the level *p* < 0.01 based on a two-sample *t*-test. The fact that the BNP and SIP curves of the AD group are not as steep as those of the NC group may mean that the information diffusion speed in the AD group is slow. Because the persistent homology-based features measure the topology of the whole brain network, it is reasonable to speculate that the slow convergence rate is caused by the reduction of functional integration of the whole brain, which may further lead to cognitive defects in patients with AD.

Further, we apply the traditional method of cluster analysis to verify the abovementioned assumption. The spatiotemporal networks of AD and NC individuals were clustered into two states. One shows the pattern with a stronger connectivity in the brain region, and another presents the pattern with less connection definitions and a lower connectivity. Moreover, the brain network of patients with AD were more likely to stay in a weak connection state. This finding confirms the abovementioned assumption about the weak integration ability of AD brain network. Therefore, our proposed method based on persistent homology provides a novel viewpoint of the overall tissue injury and the interruption of neuronal integration in patients with AD dementia.

### Clinical Potential of Spatiotemporal Brain Network

We proposed a novel method to construct and measure a spatiotemporal network based on persistent homology (i.e., spatiotemporal network + persistent homology-based properties). We compared our experimental results with two dynamic spatiotemporal methods and two static spatial methods for a functional connectivity analysis, including spatiotemporal network + graph theory-based properties, spatiotemporal network + *k*-means clustering, spatial network + persistent homology-based properties, and spatial network + graph theory-based properties. Because the strength of functional connectivity varies in the range of seconds to minutes, it is important to analyze the differences between different time periods, forming temporal dynamics. Only a spatiotemporal brain network is able to capture the inner dynamic nature of connectivity alterations, therefore the dynamic spatiotemporal methods are demonstrated to be more powerful to distinguish the AD group from the NC group than the static spatial methods without time-varying information, as shown in Tables 2, 3.

Further, in dynamic spatiotemporal network analyses, after sliding time windows, the state extraction through *k*-means clustering and the network topology measurement have been the two most widely applied strategies (Preti et al., 2017). The former technique enables to detect differences between AD and NC groups based on the dynamical occurrence and connectivity strength of connectivity states. Then, the latter technique allows to refine the meaning of the observed spatial differences across groups using some network metrics. With graph theory-based network properties, various non-trivial topological features, including small-world organization, modular structure, and highly connected hubs, have been observed to be disrupted in patients with AD (Sporns, 2018; Kuang et al., 2019a). Moreover, the persistent homology-based network properties allow to address dynamic abnormalities at a more global level, where the evolution of the global pattern of connectivity contributed by all scales is probed.

In summary, the dynamic spatiotemporal network analysis provides more completed functional alterations in connectivity, which cannot be depicted by a stationary analysis. Such kinds of technique have a greater potential for clinical applications in AD.

### Limitation and Future Works

Although the promising results were obtained by applying the two suggested network properties SIP and BNP based on persistent homology to discriminate spatiotemporal networks between AD and NC groups, there are some important caveats. First, most network properties showed a better statistical power in spatiotemporal networks in ADNI data set. To validate the robustness of the proposed method, we will further study on other independent data sets. Then, current research is limited to the analysis of the rs-fMRI data collected in a single time period and does not verify multiple time periods. In the future, we will study the longitudinal trajectories (Dautricourt et al., 2021) of functional brain dynamics over multiple time periods. Last but not least, although this article interprets the temporal dynamics of the functional brain network well and has obtained good experimental results, a correlation analysis with behavioral data should be performed in the future.

## Conclusion

This work measured the functional brain network structure of the whole brain network and its subnetwork DMN on rs-fMRI data set. A novel method is proposed based on our prior work of persistent homology. We combine multiple temporal windows and spatial scales to study the spatiotemporal brain dynamics. Most network properties show a better statistical power in spatiotemporal networks than in spatial networks, and the persistent homology-based features detected more significant differences between AD and NC groups than the standard graph theory properties. Moreover, the brain network of patients with AD is more likely to stay in a weak connection state in a clustering study. To the best of our knowledge, this is the first study applying persistent homology to analyze the spatiotemporal brain network. This study offers a novel insight into revealing the abnormalities of the functional organization of patients with AD.

## Data Availability Statement

Publicly available datasets were analyzed in this study. This data can be found here: http://adni.loni.usc.edu.

## Ethics Statement

Written informed consent was obtained from the individual(s) for the publication of any potentially identifiable images or data included in this article.

## Author Contributions

JX and LK designed the study. LK acquired the data. JX, XW, and LK analyzed and interpreted the results of the data. JX and JJ drafted the manuscript. JX and LK revised the manuscript. All authors contributed to the article and approved the submitted version.

## Conflict of Interest

The authors declare that the research was conducted in the absence of any commercial or financial relationships that could be construed as a potential conflict of interest.

## Publisher’s Note

All claims expressed in this article are solely those of the authors and do not necessarily represent those of their affiliated organizations, or those of the publisher, the editors and the reviewers. Any product that may be evaluated in this article, or claim that may be made by its manufacturer, is not guaranteed or endorsed by the publisher.

## Footnotes

1
adni.loni.usc.edu

2
http://www.fil.ion.ucl.ac.uk/spm/

3
http://restfmri.net/forum/DPARSF

4
https://sites.google.com/site/bctnet/

5
http://adni.loni.usc.edu

## References

[B1] Allen E. A. Damaraju E. Plis S. M. Erhardt E. B. Eichele T. Calhoun V. D. (2014). Tracking whole-brain connectivity dynamics in the resting state. *Cereb. Cortex* 24 663–676. 23146964 10.1093/cercor/bhs352 PMC3920766

[B2] Binnewijzend M. A. A. Adriaanse S. M. Van der Flier W. M. Teunissen C. E. de Munck J. C. Stam C. J. (2014). Brain network alterations in Alzheimer’s disease measured by Eigenvector centrality in fMRI are related to cognition and CSF biomarkers. *Hum. Brain Mapp.* 35 2383–2393. 10.1002/hbm.22335 24039033 PMC6869112

[B3] Brier M. R. Thomas J. B. Fagan A. M. Hassenstab J. Holtzman D. M. Benzinger T. L. (2014). Functional connectivity and graph theory in preclinical Alzheimer’s disease. *Neurobiol. Aging* 35 757–768. 24216223 10.1016/j.neurobiolaging.2013.10.081 PMC3880636

[B4] Chen Q. Lu J. M. Zhang X. Sun Y. Chen W. Q. Li X. (2021). Alterations in dynamic functional connectivity in individuals with subjective cognitive decline. *Front. Aging Neurosci.* 13:646017. 10.3389/fnagi.2021.646017 33613274 PMC7886811

[B5] Chen X. B. Zhang H. Gao Y. Wee C. Y. Li G. Shen D. G. (2016). High-Order resting-state functional connectivity network for MCI classification. *Hum. Brain Mapp.* 37 3282–3296. 10.1002/hbm.23240 27144538 PMC4980261

[B6] Choi H. Kim Y. K. Kang H. Lee H. Im H.-J. Kim E. E. (2014). Abnormal metabolic connectivity in the pilocarpine-induced epilepsy rat model: a multiscale network analysis based on persistent homology. *NeuroImage* 99 226–236. 24857713 10.1016/j.neuroimage.2014.05.039

[B7] Dautricourt S. de Flores R. Landeau B. Poisnel G. Vanhoutte M. Delcroix N. (2021). Longitudinal changes in hippocampal network connectivity in Alzheimer’s disease. *Ann. Neurol.* 90 391–406. 10.1002/ana.26168 34279043 PMC9291910

[B8] De Pasquale F. Della Penna S. Sporns O. Romani G. Corbetta M. (2016). A dynamic core network and global efficiency in the resting human brain. *Cereb. Cortex* 26 4015–4033. 26347485 10.1093/cercor/bhv185 PMC5027996

[B9] de Vos F. Koini M. Schouten T. M. Seiler S. van der Grond J. Lechner A. (2018). A comprehensive analysis of resting state fMRI measures to classify individual patients with Alzheimer’s disease. *Neuroimage* 167 62–72. 10.1016/j.neuroimage.2017.11.025 29155080

[B10] Edelsbrunner H. Harer J. (2010). *Computational Topology: an Introduction.* Providence, RI: American Mathematical society.

[B11] Engels M. M. A. van der Flier W. M. Stam C. J. Hillebrand A. Scheltens P. van Straaten E. C. W. (2017). Alzheimer’s disease: the state of the art in resting-state magnetoencephalography. *Clin. Neurophysiol.* 128 1426–1437. 10.1016/j.clinph.2017.05.012 28622527

[B12] Folstein M. F. Folstein S. E. McHugh P. R. (1975). “Mini-mental state”: a practical method for grading the cognitive state of patients for the clinician. *J. Psychiatric Res.* 12 189–198. 10.1016/0022-3956(75)90026-6 1202204

[B13] Fujita A. Takahashi D. Y. Balardin J. B. Vidal M. C. Sato J. R. (2017). Correlation between graphs with an application to brain network analysis. *Comput. Stat. Data Anal.* 109 76–92. 10.1016/j.csda.2016.11.016

[B14] Giusti C. Ghrist R. Bassett D. S. (2016). Two’s company, three (or more) is a simplex : algebraic-topological tools for understanding higher-order structure in neural data. *J. Comput. Neurosci.* 41 1–14. 10.1007/s10827-016-0608-6 27287487 PMC4927616

[B15] Hallquist M. N. Hillary F. G. (2018). Graph theory approaches to functional network organization in brain disorders: a critique for a brave new small-world. *Network Neurosci.* 3 1–26. 10.1162/netn_a_00054 PMC6326733 30793071

[B16] Jack C. R. Bernstein M. A. Fox N. C. Thompson P. Alexander G. Harvey D. (2008). The Alzheimer’s disease neuroimaging initiative (ADNI): MRI methods. *J. Magn. Reson. Imaging* 27 685–691. 18302232 10.1002/jmri.21049 PMC2544629

[B17] Jagust W. J. Bandy D. Chen K. Foster N. L. Landau S. M. Mathis C. A. (2010). The Alzheimer’s disease neuroimaging initiative positron emission tomography core. *Alzheimer’s Dementia* 6 221–229. 10.1016/j.jalz.2010.03.003 PMC2920531 20451870

[B18] Kuang L. Han X. Chen K. (2019a). A concise and persistent feature to study brain resting-state network dynamics: findings from the Alzheimer’s disease Neuroimaging Initiative. *Hum. Brain Mapp.* 40 1062–1081. 10.1002/hbm.24383 30569583 PMC6570412

[B19] Kuang L. Zhao D. Xing J. Chen Z. Xiong F. Han X. (2019b). Metabolic Brain Network Analysis of FDG-PET in Alzheimer’s disease using kernel-based persistent features. *Molecules* 24:2301. 10.3390/molecules24122301 31234358 PMC6630461

[B20] Kuang L. Q. Gao Y. Chen Z. Y. Xing J. C. Xiong F. G. Han X. (2020a). White matter brain network research in Alzheimer’s disease using persistent features. *Molecules* 25:2472. 10.3390/molecules25112472 32471036 PMC7321261

[B21] Kuang L. Q. Jia J. Y. Zhao D. Y. Xiong F. G. Han X. Wang Y. L. (2020b). Default mode network analysis of APOE genotype in cognitively unimpaired subjects based on persistent homology. *Front. Aging Neurosci.* 12:188. 10.3389/fnagi.2020.00188 32733231 PMC7358981

[B22] Lee H. Kang H. Chung M. K. Kim B.-N. Lee D. S. (2012). Persistent brain network homology from the perspective of dendrogram. *IEEE Trans. Med. Imaging* 31 2267–2277. 23008247 10.1109/TMI.2012.2219590

[B23] Lee H. Kang H. Chung M. K. Lim S. Kim B. N. Lee D. S. (2017). Integrated multimodal network approach to PET and MRI based on multidimensional persistent homology. *Hum. Brain Mapp.* 38 1387–1402. 27859919 10.1002/hbm.23461 PMC6867151

[B24] Lei B. Y. Yu S. Z. Zhao X. Frangi A. F. Tan E. L. Elazab A. (2021). Diagnosis of early Alzheimer’s disease based on dynamic high order networks. *Brain Imaging Behav.* 15 276–287. 10.1007/s11682-019-00255-9 32789620

[B25] Liao W. Wu G. R. Qiang X. Ji G. J. Zhang Z. Zang Y. F. (2014). DynamicBC: a MATLAB toolbox for dynamic brain connectome analysis. *Brain Connect.* 4:780. 25083734 10.1089/brain.2014.0253 PMC4268585

[B26] Marquez F. Yassa M. A. (2019). Neuroimaging biomarkers for Alzheimer’s disease. *Mol. Neurodegener.* 14:21. 10.1186/s13024-019-0325-5 31174557 PMC6555939

[B27] Mill R. D. Gordon B. A. Balota D. A. Cole M. W. (2020). Predicting dysfunctional age-related task activations from resting-state network alterations. *Neuroimage* 221:117167. 10.1016/j.neuroimage.2020.117167 32682094 PMC7810059

[B28] Patterson C. (2018). *World Alzheimer Report 2018.* London: Alzheimer’s Disease International.

[B29] Preti M. G. Bolton T. A. W. Van De Ville D. (2017). The dynamic functional connectome: State-of-the-art and perspectives. *Neuroimage* 160 41–54. 10.1016/j.neuroimage.2016.12.061 28034766

[B30] Rauchmann B.-S. Ersoezlue E. Stoecklein S. Keeser D. Brosseron F. Buerger K. (2021). Resting-State network alterations differ between Alzheimer’s disease atrophy subtypes. *Cereb. Cortex* 31 4901–4915. 10.1093/cercor/bhab130 34080613 PMC8491689

[B31] Rubinov M. Sporns O. (2010). Complex network measures of brain connectivity: uses and interpretations. *NeuroImage* 52 1059–1069. 19819337 10.1016/j.neuroimage.2009.10.003

[B32] Scheltens P. De Strooper B. Kivipelto M. Holstege H. Chetelat G. Teunissen C. E. (2021). Alzheimer’s disease. *Lancet* 397 1577–1590. 10.1016/s0140-6736(20)32205-4 33667416 PMC8354300

[B33] Smith S. M. Nichols T. E. (2009). Threshold-free cluster enhancement: addressing problems of smoothing, threshold dependence and localisation in cluster inference. *Neuroimage* 44 83–98. 10.1016/j.neuroimage.2008.03.061 18501637

[B34] Sporns O. (2018). Graph theory methods: applications in brain networks. *Dial. Clin. Neurosci.* 20 111–121. 10.31887/DCNS.2018.20.2/osporns PMC6136126 30250388

[B35] Tzourio-Mazoyer N. Landeau B. Papathanassiou D. Crivello F. Etard O. Delcroix N. (2002). Automated anatomical labeling of activations in SPM using a macroscopic anatomical parcellation of the MNI MRI single-subject brain. *NeuroImage* 15 273–289. 11771995 10.1006/nimg.2001.0978

[B36] Vriend C. van den Heuvel O. A. Berendse H. W. van der Werf Y. D. Douw L. (2018). Global and subnetwork changes of the structural connectome in de novo Parkinson’s disease. *Neuroscience* 386 295–308. 10.1016/j.neuroscience.2018.06.050 30004009

[B37] Wang J. Zuo X. Dai Z. Xia M. Zhao Z. Zhao X. (2013). Disrupted functional brain connectome in individuals at risk for Alzheimer’s disease. *Biol. Psychiatry* 73 472–481. 10.1016/j.biopsych.2012.03.026 22537793

[B38] Watts D. J. Strogatz S. H. (1998). Collective dynamics of ‘small-world’ networks. *Nature* 393:440. 9623998 10.1038/30918

[B39] Yan C. Zang Y. (2010). DPARSF: a MATLAB toolbox for” pipeline” data analysis of resting-state fMRI. *Front. Sys. Neurosci.* 4:13. 10.3389/fnsys.2010.00013 20577591 PMC2889691

